# Asymmetric optical cryptosystem for multiple images based on devil’s spiral Fresnel lens phase and random spiral transform in gyrator domain

**DOI:** 10.1038/s41598-021-00276-9

**Published:** 2021-10-21

**Authors:** Hang Chen, Zhengjun Liu, Camel Tanougast, Walter Blondel

**Affiliations:** 1 School of Space Information, Space Engineering University, Beijing, 101416 China; 2 CNRS, CRAN UMR 7039, Université de Lorraine, 54000 Nancy, France; 3 Laboratoire Conception Optimisation et Modélisation des Systèmes, Université de Lorraine, 57070 Metz, France; 4 Department of Automation Measurement and Control, Harbin Institute of Technology, Harbin, 150001 China

**Keywords:** Imaging and sensing, Displays, Optoelectronic devices and components

## Abstract

An asymmetric cryptosystem is presented for encrypting multiple images in gyrator transform domains. In the encryption approach, the devil’s spiral Fresnel lens variable pure phase mask is first designed for each image band to be encrypted by using devil’ mask, random spiral phase and Fresnel mask, respectively. Subsequently, a novel random devil’ spiral Fresnel transform in optical gyrator transform is implemented to achieved the intermediate output. Then, the intermediate data is divided into two masks by employing random modulus decomposition in the asymmetric process. Finally, a random permutation matrix is utilized to obtain the ciphertext of the intact algorithm. For the decryption approach, two divided masks (private key and ciphertext) need to be imported into the optical gyrator input plane simultaneously. Some numerical experiments are given to verify the effectiveness and capability of this asymmetric cryptosystem.

## Introduction

The secure information storage and transmission techniques have attracted increasingly attention with the rapid development of multimedia communication and internet technology. Optical information security techniques is one of the most hottest way to encrypt/decrypt the secret information due to its unique superiority of high-speed calculation, parallel processing capability and multiple degrees of freedom design space. The first optical encryption system named double random phase encoding (DRPE) is designed by Refrégiér and Javidi^1^, which is a $4f$ system composed by two Fourier lenses. Subsequently, some optical encryption cryptosystem based on DRPE are reported^2–4^. However, the DRPE is verified as vulnerable to some potential attack algorithm due to the linearity property^5–7^. To break the linear relationship and enhance the security of the optical encryption system, various optical transform have been employed to design the high security optical encryption systems, such as optical gyrator transform^8,9^, fractional Fourier transform^10,11^, Fresnel transform^2,12,13^. Furthermore, some optical techniques, such as polarized light interference^14,15^ and phase retrieval algorithm^16,17^, are also considered and utilized to improve the security level of the optical cryptosystem.

In 2005, Situ and Zhang first proposed a multi-image cryptosystem based on wavelength multiplexing technology^1^. The multiple image encryption idea soon attracts great attention owing to its economic memory occupation and high efficiency transmission^18–24^. Basically, most of these presented schemes are symmetric encryption algorithms, which use the identical key both in encryption and decryption process. In fact, the DRPE-based optical cryptosystem has been verified as vulnerable to some potential attack algorithms because of its symmetrical key system^24^. Recently, some optical asymmetric encryption schemes for multiple images have been reported and the corresponding virtual optical encryption systems have verified the validity of the asymmetric cryptosystem^25–29^. However, most of these multiple image encryption schemes are designed for specific number of images, eight images^28^, for instance. The optical cryptosystem for variable number of images have not been deeply researched for the best of our knowledge.

In this paper, an asymmetric optical encryption algorithm for variable number of images using random devil’ spiral Fresnel transform in optical gyrator transform is proposed. The random devil’s spiral Fresnel lens (DSFs) phase is considered and utilized for encoding the different band of the multiple image cube first. Subsequently, the encoded data are transformed by random devil’ spiral Fresnel transform (RDST) in optical gyrator light filed. The intermediate data recorded by charge coupled device (CCD) then decomposed into two masks by using random modulus decomposition (RMD) in the asymmetric process. Finally, the final ciphertext is obtained by scrambling with a random permutation matrix, which can enhance the security. At the aspect of decryption, both the ciphertext and private key are necessary to be imported into the optical gyrator input plane simultaneously. The proposed cryptosystem is suitable to protect the massive remote sensing image, for instance, remote sensing hypersepctral image. Various numerical experiments are performed to validate the performance of the proposed asymmetric cryptosystem.

The rest of this paper is arranged in the following sequence. In "Optical asymmetric cryptosystem" section, the intact asymmetric cryptosystem is presented in detail. In "Numerical simulation" section, various experiments results are given to demonstrate the validity and capability of the algorithm. The brief conclusion is drawn in the final section.

## Optical asymmetric cryptosystem

In this section, the random devil’s spiral Fresnel lens (DSFs) phase, random devil’ spiral Fresnel transform (RDST), random modulus decomposition and the random permutation matrix are introduced individually. Thereafter, the corresponding hybrid optoelectronic architectures of the proposed encryption/decryption algorithm are presented. Finally, the intact optical asymmetric encryption scheme is addressed in detail.

### Random devil’s spiral Fresnel lens (DSFs) phase

Referring to^30,31^, the phase function of devil’s lens can be expressed by one dimensional Cantor function, which can be regards as one particular kind of devil’s staircase. In the domain [0, 1], the triadic Cantor function can be written as follows

1 $$ F_{S} (x) = \left\{ {\begin{array}{*{20}l} {\frac{l}{{2^{S} }}{\kern 1pt} } \hfill & {if\quad p_{S,l} \le x \le q_{S,l} } \hfill \\ {\frac{1}{{2^{S} }}\frac{{x - q_{S,l} }}{{p_{S,l + 1} - q_{S,l} }} + \frac{1}{{2^{S} }}} \hfill & {if\quad q_{S,l} \le x \le p_{S,l + 1} } \hfill \\ \end{array} } \right. $$

where the parameters $p_{S,l + 1}$ and $q_{S,l}$ are two threshold to control the step of the devil’s staircase. In this paper, the parameter $S$ is set as 3. Therefore, the step of devil’s staircase will be defined in the interval $p_{3,l} \le x \le q_{3,l}$ ($l = 1,\ldots,7$).

According to the triadic Cantor function described above, the devil’s lens can be expressed as

2 $$ D_{S} (\zeta ) = \exp ( - i2^{S + 1} \pi F_{S} ) $$

where $F_{S}$ represent the triadic Cantor function. The input element $\zeta = \left( \frac{r}{a} \right)^{2}$ can be regarded as the normalized quadratic radial coordinate, where the coefficient $a$ denotes the radius of the lens and $r = \sqrt {x^{2} + y^{2} }$. In fact, the devil’s lens is a diffractive optical element create circularly symmetric pure phase.

In addition, the pure quadratic phase function from Fresnel lens can be defined as

3 $$ FE_{{\lambda ,f_{0} }} (\zeta ) = \exp \left( {\frac{{i\pi (r)^{2} }}{{\lambda f_{0} }}} \right) $$

where the parameters $\lambda$ and $f_{0}$ represent the wavelength of the incident light and focal length, respectively. Here, the parameters $\lambda$ and $f_{0}$ are taken 632.8 nm and 900 mm in calculation.

The spiral phase function (SPF) is one kind of signum function, which can be used for two-dimensional Hilbert transform^32,33^. To design the random devil’s spiral Fresnel lens (DSFs) phase, an improved random spiral phase function in two dimensional domains is introduced as follows^34^

4 $$ \left\{ {\begin{array}{*{20}l} {SPF(x,y) = \frac{x + iy}{{\sqrt {x^{2} + y^{2} } }} = \exp (i \cdot \varphi (x,y))} \hfill \\ {RSPF(x,y) = \exp (i \cdot p \cdot \varphi (x,y))} \hfill \\ \end{array} } \right. $$

where $(x,y)$ and $\varphi (x,y)$ in the function spiral phase function denote the spatial coordinates and frequency domain polar angle, respectively. The improved spiral phase function (RSPF) is designed by introducing a random parameter $p$ to control the number of singularities.

**Figure 1:**
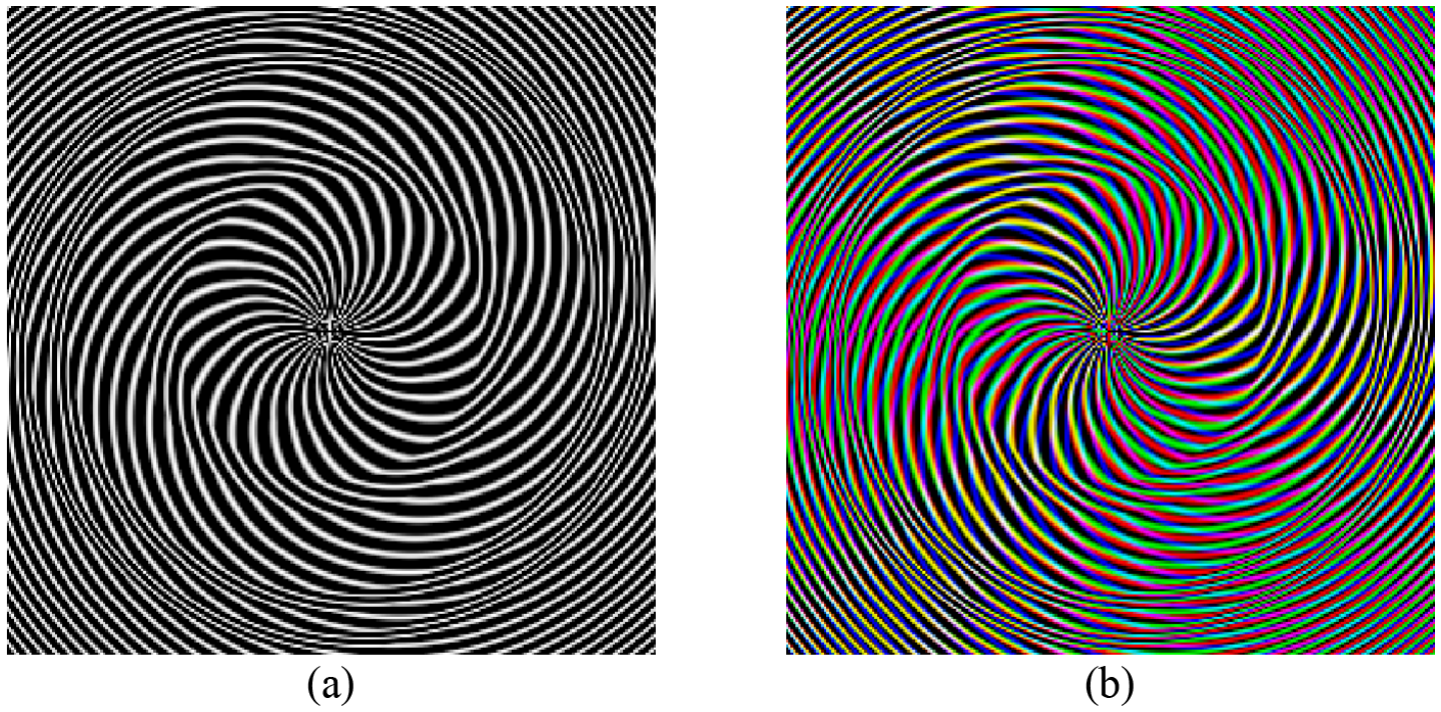
The DSFs phase function (**a**) gray version and (**b**) RGB color version.

Based on the devil’s lens function, pure quadratic phase function of Fresnel lens and random spiral phase function introduced in Eqs. 1–4, the random devil’s spiral Fresnel lens (DSFs) phase can be defined as

5 $$ DSF_{S} = \exp \left( { - i2^{S + 1} \pi F_{S} + \frac{{i\pi (r)^{2} }}{{\lambda f_{0} }}} \right) + i \cdot p \cdot \varphi (x,y) $$

where the parameter $r = \sqrt {x^{2} + y^{2} }$, which indicate the polar length in frequency space. It can be seen that the DSFs phase mask can be obtained by combining the phase functions of $D_{S} (\zeta )$, $FE_{{\lambda ,f_{0} }} (\zeta )$ and $RSPF$. Therefore, the parameters in these functions can be regarded as the extra keys to protect the secret image. Some DSFs phase masks in color format with specific parameters $S = 3$ and $p = (30,28,49)$ are illustrated in Fig. 1.

### Random devil’ spiral Fresnel transform (RDST) in Gyrator domain

The optical gyrator transform is first proposed by Simon and its feasibility has been verified by using a six lenses optical system in 2007^35,36^. In particular, the gyrator transform has only two-dimensional version and one rotation angle parameter is introduced in the transform, which can be regarded as the fractional order of the gyrator transform. For a input two-dimensional image $f(x,y)$, the mathematical definition of the gyrator transform can be written as

6 $$ \begin{aligned} G(u,v) = & \xi^{\alpha } [f(x,y)](u,v) \\ = & \frac{1}{{\left| {\sin \theta } \right|}}\iint {f(x,y)}\exp [i2\pi \frac{{\left( {xy{ + }uv} \right){\text{cos}}\theta - xv - yu}}{\sin \theta }]{\text{d}}x{\text{d}}y, \\ \end{aligned} $$

where $f(x,y)$ and $G(u,v)$ are the input and output function of the gyrator transform, respectively. The parameter $\alpha$ represents the fractional order, which obeys the properties of index additivity and linearity. In particular, the gyrator transform equal to the traditional Fourier transform when $\alpha = \, \pi /{2}$. The invers version of gyrator transform $\xi^{\alpha }$ is $\xi^{{{ - }\alpha }}$ or $\xi^{{2\pi { - }\alpha }}$. The gyrator transform is considered and utilized to complete the random devil’ spiral Fresnel transform in the following step. In theory, other transformations, like fractional Fourier transform or Hartley transform, are also can be employed.

**Figure 2:**
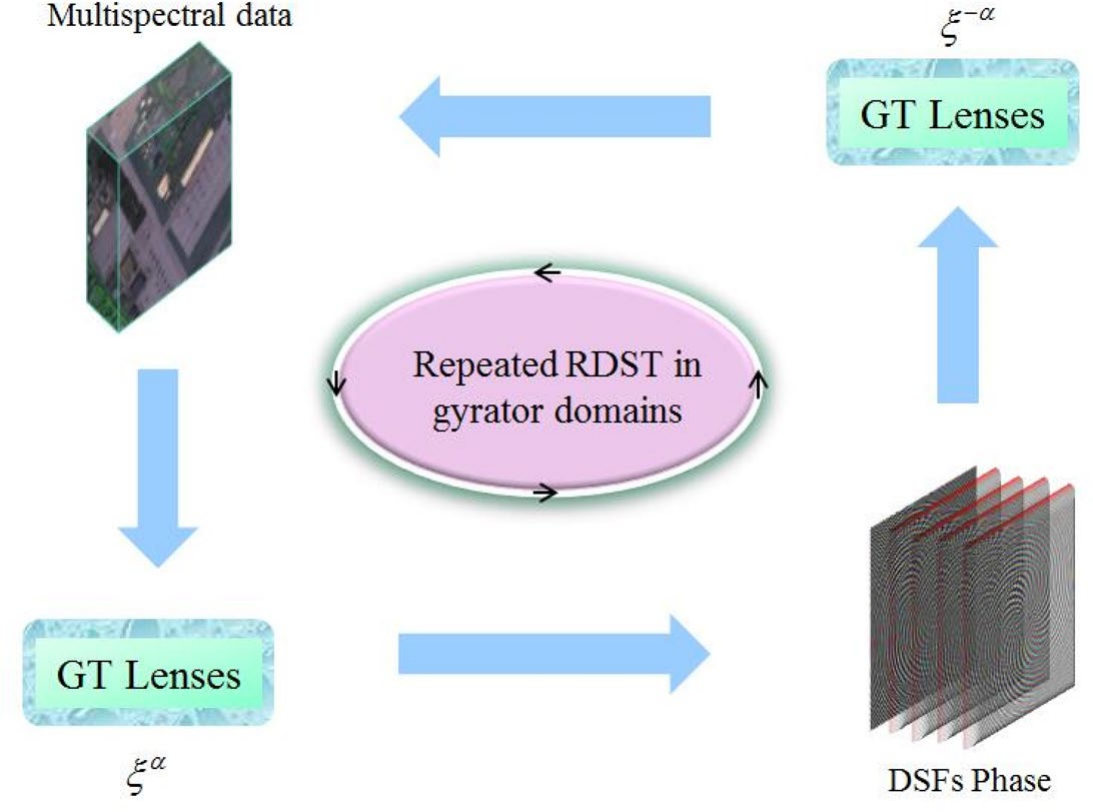
The schematic diagram of random devil’ spiral Fresnel transform.

Based on the random devil’s spiral Fresnel lens (DSFs) phase described in "Random devil’s spiral Fresnel lens (DSFs) phase" section, we present a random devil’ spiral Fresnel transform (RDST) in optical Gyrator domain. Suppose $I(x,y)$ is the original secret image to be encrypted, the random devil’s spiral Fresnel transform can be defined as follows

7 $$ RDST(I(x,y)) = \xi^{ - \alpha } \left\{ {DSFs.\xi^{\alpha } [I(x,y)](u,v)} \right\}(u,v) $$

where the functions $\xi^{\alpha }$ and $\xi^{ - \alpha }$ denote the gyrator transform and its inverse transform, which means the optical gyrator transform is performed two times in random devil’s spiral Fresnel transform. The inverse RDST is given by

8 $$ IRDST(I(x,y)) = \xi^{ - \alpha } \left\{ {conj(DSFs).\xi^{\alpha } [I(x,y)](u,v)} \right\}(u,v) $$

where the symbol ‘conj’ indicates the calculate the conjugation of RDST. The schematic diagram of Random devil’ spiral Fresnel transform in optical Gyrator domain is illustrated in Fig. 2. Both the RDST and its inverse transform IRDST can be implemented in a hybrid optoelectronic architecture system, which composed by optical gyrator lenses group, spatial light modulators (SLM) and computer. Details information will be discussed in "The asymmetric cryptosystem for multiple images" section.

### The random modulus decomposition

A two-dimensional complex function can be decomposed by using random modulus decomposition. In fact, the random modulus is one kind of unequal modulus decomposition. By introducing the random parameter in the decomposition algorithm, a two-dimensional complex image can be separated into two random statistically independent masks. Here, suppose $G(u,v)$ is the result of gyrator transform of two-dimensional image $I(x,y)$. Besides, the amplitude and phase function of $G(u,v)$ can be expressed by $A(u,v) = \left| {G(u,v)} \right|$ and $\varphi (u,v) = \arg [G(u,v)]$, respectively. Therefore, the mathematical definition of random modulus decomposition can be written as

9 $$ \left\{ {\begin{array}{*{20}c} {P1(u,v) = \frac{A(u,v) \cdot \sin [\beta (u,v)]}{{\sin [\delta (u,v){ + }\beta (u,v)]}}\exp \{ i[\varphi (u,v) - \delta (u,v)\} ,} \\ {P2(u,v) = \frac{A(u,v) \cdot \sin [\delta (u,v)]}{{\sin [\delta (u,v) + \beta (u,v)]}}\exp \{ i[\varphi (u,v) + \beta (u,v)]\} .} \\ \end{array} } \right. $$

where the function $\delta (u,v)$ and $\beta (u,v)$ represents the random function calculated by $\alpha (u,v) = 2\pi * rand(u,v)$ and $\varphi (u,v) = 2\pi * rand(u,v)$, which are the random function distributed uniformly in the domain of $[0,\;2\pi ]$. Note that the random modulus decomposition reduces the constraints efficiently and leads to the effective trapdoor one-way function.

### The asymmetric cryptosystem for multiple images

**Figure 3:**
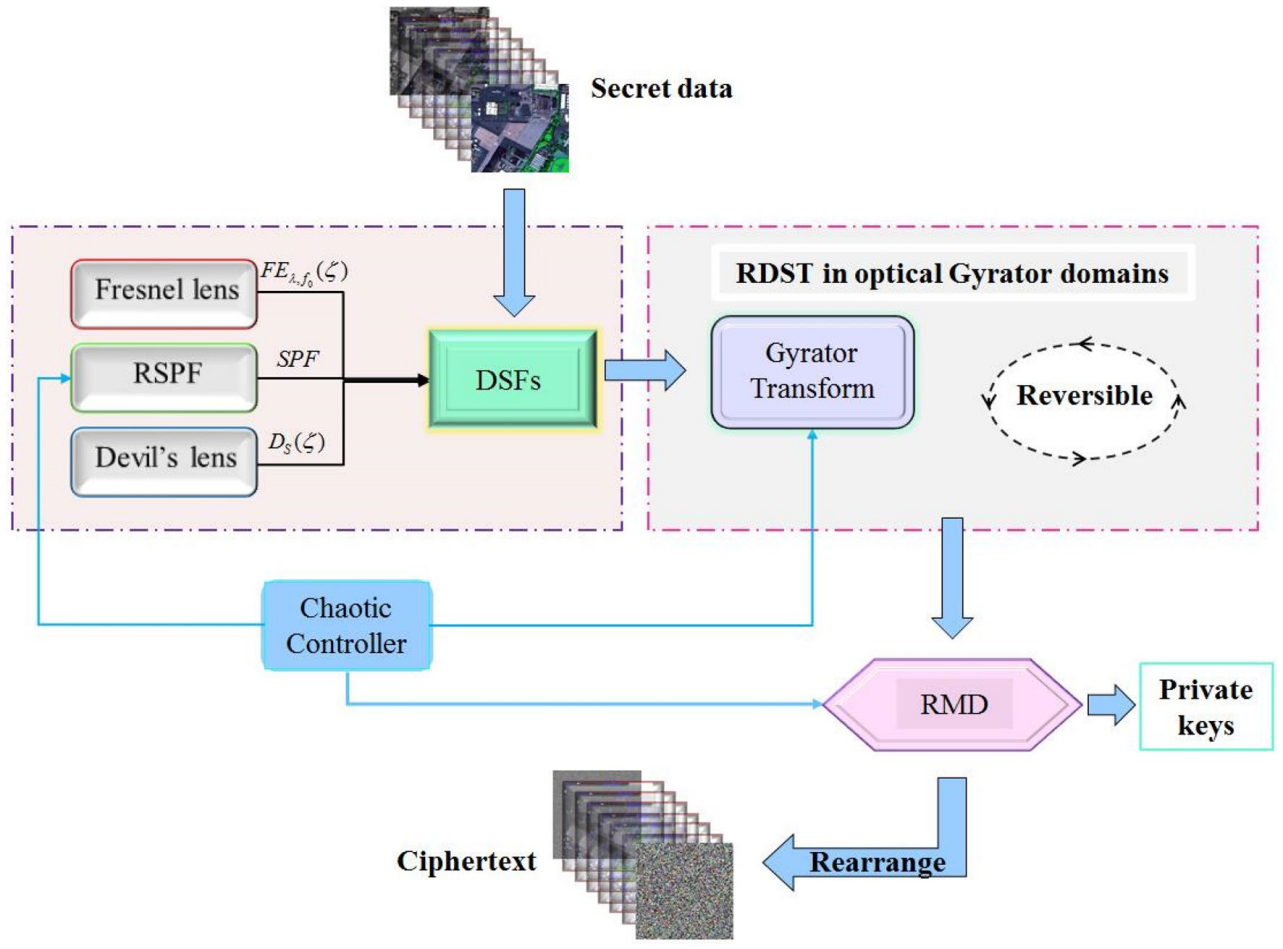
The flowchart of the proposed cryptosystem for multiple images.

According to the flowchart of the intact proposed cryptosystem depicted in Fig. 3, the multispectral image is divided into several single bands before the calculation. First of all, the circularly symmetric pure phase $D_{S} (\zeta )$ and pure quadratic phase $FE_{{\lambda ,f_{0} }} (\zeta )$ are created by the devil’s lens and Fresnel lens, respectively. By combining the phase functions of $D_{S} (\zeta )$, $FE_{{\lambda ,f_{0} }} (\zeta )$ and $RSPF$, the DSFs phase mask can be obtained as shown in Fig. 1. Subsequently, the single band image and DSFs phase mask are merged randomly and imported into the random devil’ spiral Fresnel transform in Gyrator domain simultaneously. To enhance security of the algorithm, the fractional order $\alpha$ is randomly changed in the optical plain.

The output function of RDST then can be regarded as the input data of RMD, in which the intermediate data is decomposed into two random statistically independent masks $P1$ and $P2$. Note that the output function of RMD is complex image since the output of optical gyrator transform is complex data. In the asymmetric process, the decomposed mask $P1$ serves as the private key, while the other mask $P2$ is scrambled by using a random permutation matrix and the rearranged mask is the final ciphertext of the proposed encryption algorithm. For the decryption process, both the private key and ciphertext are necessary in the decryption input plain. The private key $P1$ and repaired mask $P2$ interfere in a beam splitter (BS) by using two SLM independently. Thereafter, the secret multispectral image can be decrypted by implemented inverse random devil’ spiral Fresnel transform in gyrator domain. In addition, some parameters, such as fractional order $\theta$ in gyrator transform and the wavelength of the incident light $\lambda$, serve as the extra keys of the cryptosystem to improve the security.

**Figure 4:**
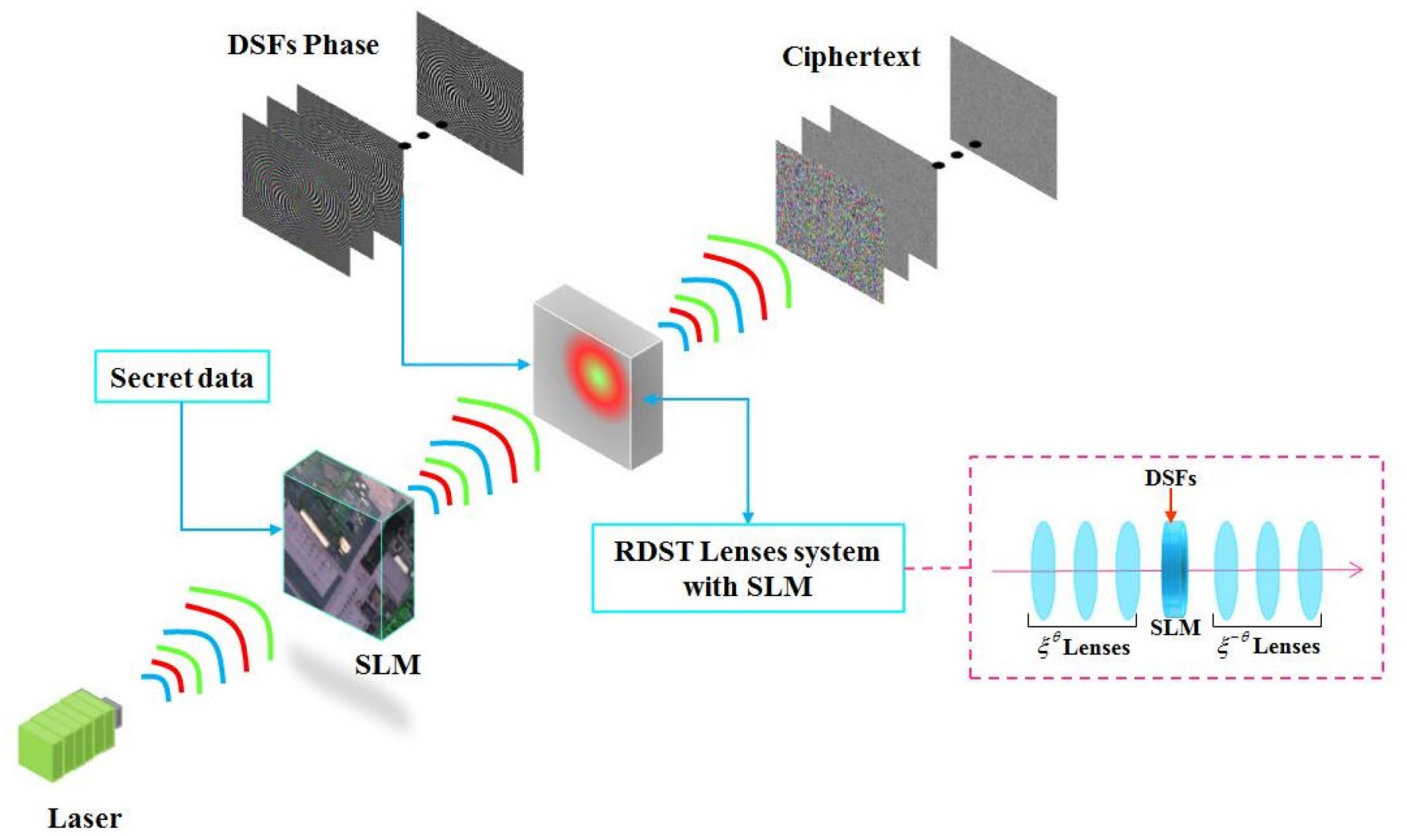
The Schematic diagram of the hybrid optoelectronic architectures.

As mentioned above, the cryptosystem can be implemented by hybrid optoelectronic architectures as shown in Fig. 4. Note that the encryption hardware setup is different from the decryption one since the proposed encryption system is asymmetric. The optical gyrator transform and inverse transform are achieved by gyrator lenses system in the optical path as displayed in Fig. 4. Some calculation, such as random modulus decomposition and random pixels permutation, are finished by computer. Note that the pure quadratic phase can be both by computer or Fresnel lens system. The data communication between optical system and computer is accomplished by spatial light modulator (SLM) and charge coupled device (CCD). At the output plain of the optoelectronic setup, the amplitudes function is recorded by CCD and the phase function can be recorded by using off-line holography techniques. Therefore, each band of the multispectral image is encrypted when the beams propagated through the hybrid electro-optical hardware-based cryptographic and then recorded and transmitted into the computer. Note that the speckle noise is not considered in the described experimental hybrid setup.

## Numerical simulation

In this section, numerical experiments are implemented to demonstrate the validity and robustness of the proposed encryption algorithm. To complete the numerical simulations in the following step, a preprocessed multispectral image ‘Sandiego’ is considered as the original secret images, in which the image size is $256 \times 256 \times 50$. As mentioned above, some parameters involved in optical gyrator transform, RDST and RMD is generated by a chaotic system, and these parameters can be regarded as the extra keys to enhance the security of the cryptosystem. In calculation, these parameters are variable for different bands of the multispectral image.

**Figure 5:**
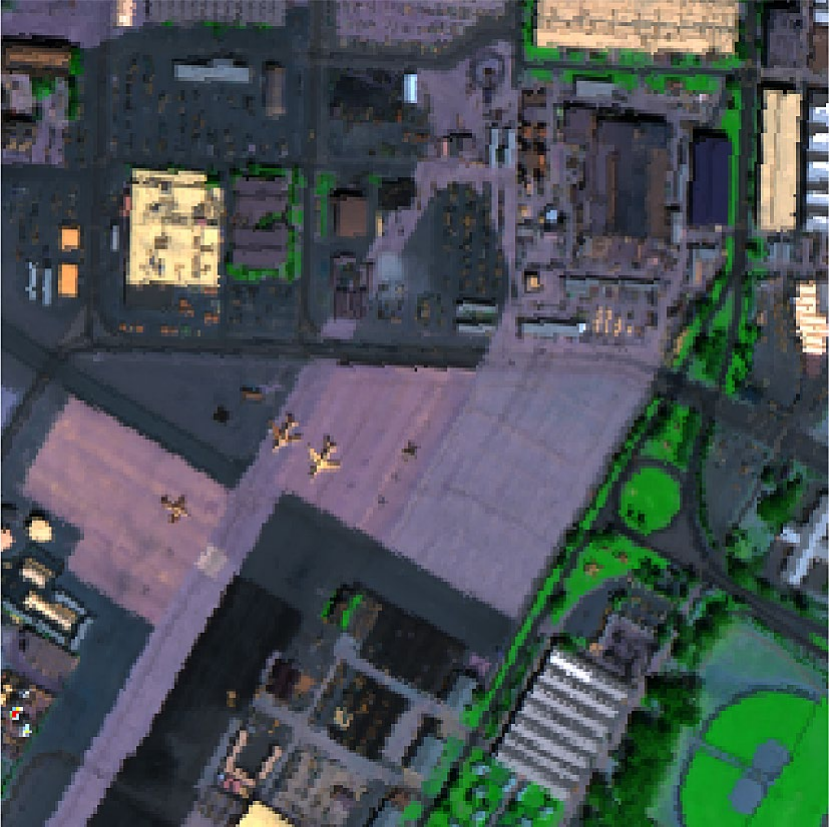
The RGB color image of the original multispectral data used in this paper.

**Figure 6:**
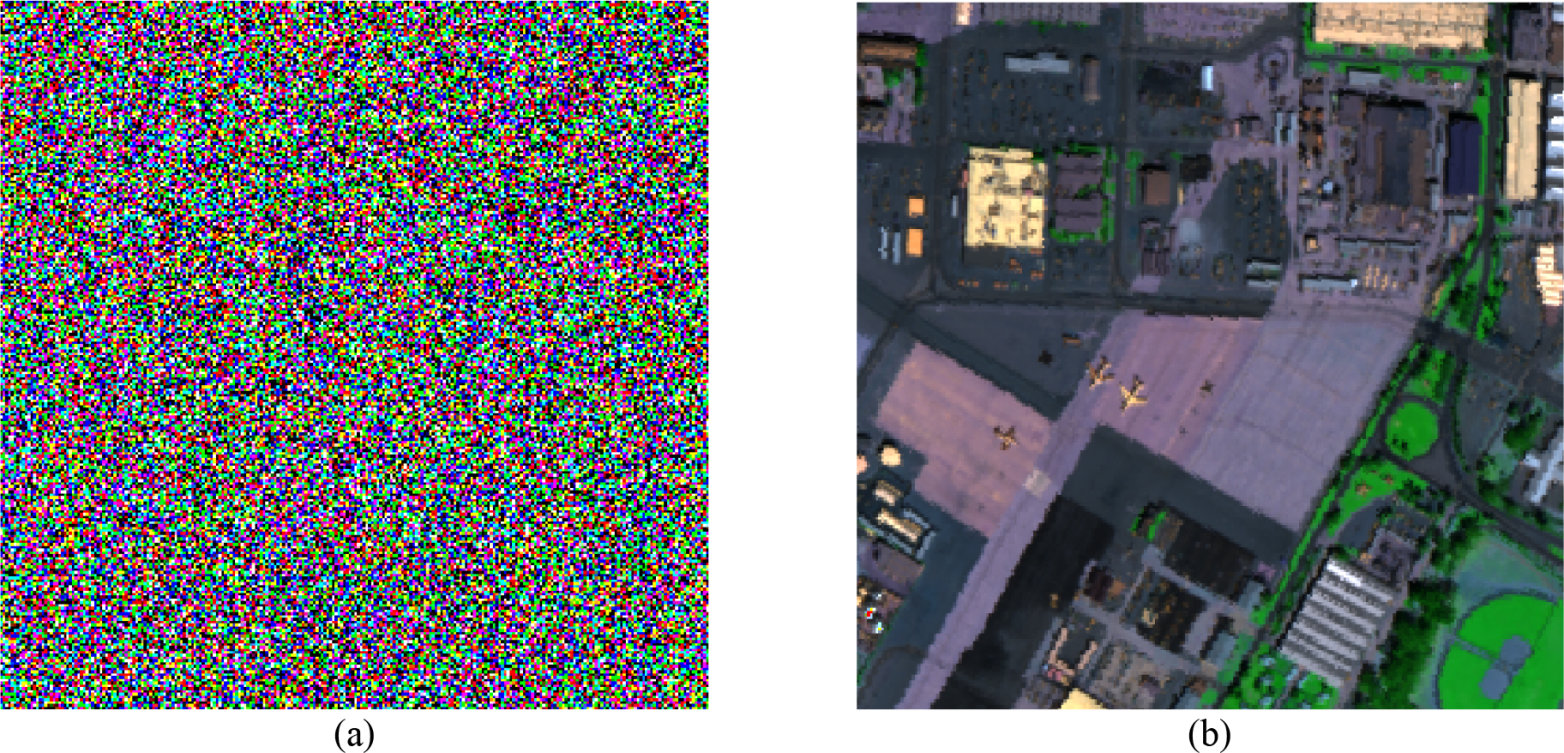
The experimental results: (**a**) the encrypted data of the pseudo color image and (**b**) the corresponding correct decrypted image.

A pseudo color image composed by the 10th, 30th and 50th band of the original multispectral data is depicted in Fig. 5. According to the complete encryption algorithm described in "Optical asymmetric cryptosystem" section, the corresponding encrypted image and the correct decrypted pattern are displayed in Fig. 6a, b, respectively. Obviously, the similarity of the original color image and the decrypted image indicates the effectiveness of the proposed cryptosystem. Note that the encrypted data and decrypted image shown in Fig. 6 are the amplitude part of the complex data achieved by the proposed encryption system. The average encryption/decryption time of each band image is 0.035 s and 0.029 s by using a computer with Core i7, CPU 2.2 GHz and 8 GB memory running Windows 7 system.

The quantitative analysis is considered to evaluate the proposed cryptosystem in the following step. First of all, some statistical measures and evaluation criterion, like peak signal-to-noise ratio (PSNR) and Normalized Cross Correlation (NCC), are introduced briefly. The PSNR can be used for estimating the difference between two images, the original image and decrypted image, for instance. The mathematical definition of PSNR can be written as follows

10 $$ PSNR(I_{{\text{d}}} ,I_{0} ) = 10\log_{10} \frac{{255^{2} M \times N}}{{\sum\limits_{\forall x,y} {\left[ {I_{{\text{d}}} (x,y) - I_{0} (x,y)} \right]^{2} } }}{\text{(dB)}}{.} $$

here the input functions $I_{{\text{d}}}$ and $I_{0}$ represent the decrypted image and original image, respectively. The symbols ‘d’ and ‘o’ are short for decrypted and original. Besides, the parameters $M$ and $N$ denote the length and width of the two input images. Generally speaking, the difference between $I_{{\text{d}}}$ and $I_{0}$ cannot be recognized by human eyes when the PSNR value larger than 50. The larger value of PSNR indicates the higher similarity between the two input images.

The other statistical measure NCC is also introduced in this section. The NCC between the plaintext and recovered image can be computed by using the equation as follows

11 $$ NCC(I_{o} ,I_{d} ) = \frac{{\sum {I_{o} (x,y) \times I_{d} (x,y)} }}{{\sqrt {\sum {I_{o} (x,y) \times I_{o} (x,y)} } \times \sqrt {\sum {I_{d} (x,y) \times I_{d} (x,y)} } }} $$

similarly, $I_{{\text{d}}}$ and $I_{0}$ represent the decrypted image and original image, respectively. The NCC value more close to 1 denotes the higher similarity between two input images. On the contrary, the lower NCC value indicates the greater dissimilarity. Note that the NCC value is fixed at the range of [0, 1].

**Figure 7:**
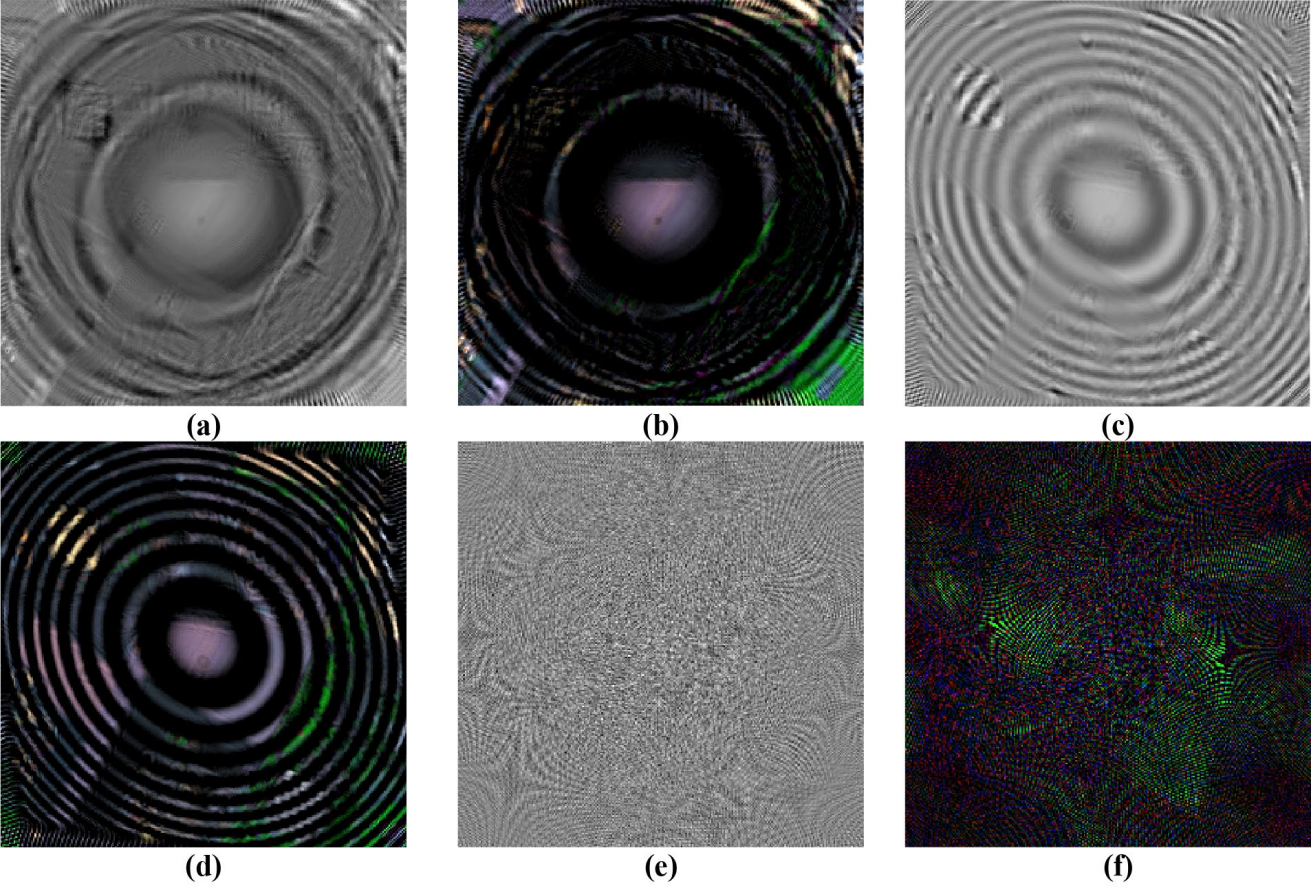
The decrypted results by using incorrect parameters in devil’s lens (**a**) single band format, (**b**) color format, incorrect focal length in Fresnel lens (**c**) single band format, (**d**) color format, incorrect $p$ in RSPF (**e**) single band format and (**f**) color format.

As discussed in "Introduction" and "Optical asymmetric cryptosystem" sections, the random devil’s spiral Fresnel lens phase is considered and utilized for encoding the different band of the multiple image. Therefore, some experiments to test the performance of the DSFs on protecting the secret information are implemented. As described in "Optical asymmetric cryptosystem" section, the DSFs is composed by using the devil’s lens, Fresnel lens and RSPF. Hence, the devil’s lens is test first. Suppose that the decryption algorithm and all the keys are known by the illegal user, but not the correct devil’s lens. The decrypted result of single 30th band and RGB color image by using the wrong coefficients $S$ and $r$ are shown in Fig. 7a, b, respectively. Obviously, most of the secret information is lost and the detail information cannot be recognized by these decrypted images. Besides, the decryption experiment by using incorrect focal length $f_{0}$ of the Fresnel lens is also implemented and the corresponding decrypted single 30th band and RGB color format image are illustrated in Fig. 7c, d, in which the detail information of the original image cannot be recognized. Finally, the chaotic data involved in RSPF is test. As shown in Fig. 3, the random order $p$ to control the number of singularities is generated by a chaotic system. In the decryption approach, the parameter $p$ is replaced by an incorrect chaotic sequence and the decrypted single 30th band and color image are displayed in Fig. 7e, f, respectively. In decryption attack, the random order $p$ for the 10th, 30th and 50th band are taken at 37.12, 4.39 and 19.88, while the correct $p$ are 33.65, 20.05 and 4.15, respectively. Apparently, the decrypted results are completely noise pattern and the secret information is well under protection of chaotic data $p$. Based on the experimental results described above, each part of the DSFs, including devil’s lens, Fresnel lens and RSPF, can protect the original multispectral image well independently. In fact, some additional numerical experiments have been implemented and the results demonstrate that the other parameters in DSFs can also be used as the extra keys to protect the input secret information.

**Figure 8:**
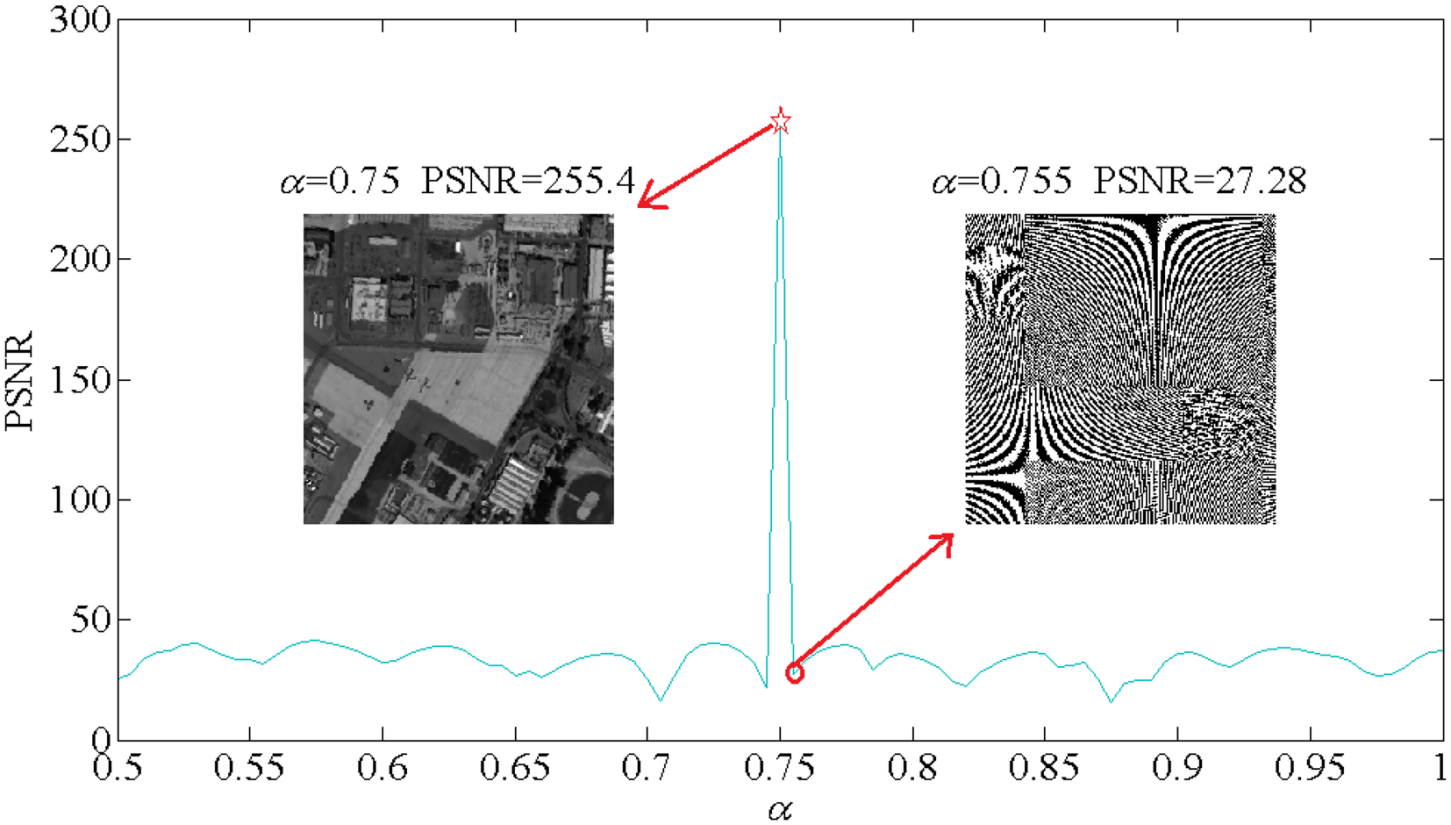
The PSNR curve between original image and attack result by using changing $\alpha$.

To test the security of the fractional order $\alpha$ in optical gyrator transform, the decryption experiment by using variable $\alpha$ is executed. Since the parameter $\alpha$ can be controlled in the propagation light path, this decryption experiment can be implemented physically by rotating the gyrator lenses system. In the encryption approach, $\alpha$ is taken as 0.75 for all the band of the multispectral image. For the attack experiment, we suppose that the attacker intercepted the private key and additional keys except $\alpha$. Therefore, the decryption process is executed for 101 times by using parameter $\alpha$ changing from 0.4 to 0.9 with step length 0.005. For the sake of simplify the calculation, only the attack results of the 30th band from the multispectral image is considered to shown. Under the circumstances described above, the PSNR curve between the original 41th band image and the corresponding attacked result is illustrated in Fig. 8, in which the PSNR curve increase sharply when the parameter $\alpha$ achieve the correct value 0.7. The sharp peak of the PSNR curve indicates the sensitivity of the parameter $\alpha$ in protecting the secret information. Moreover, the parameter $\alpha$ is generated by the chaotic system in formal encryption process, which can enhance the security of the cryptosystem. In addition, two attack results by using incorrect key $\alpha = 0.75$ and $\alpha = 0.755$ are also depicted in Fig. 8. Obviously, the attack results using incorrect $\alpha$ are almost noise pattern even $\alpha$ is very close to the real one. Therefore, the fractional order $\alpha$ severs as the important key for protecting the original secret multispectral image.

**Figure 9:**
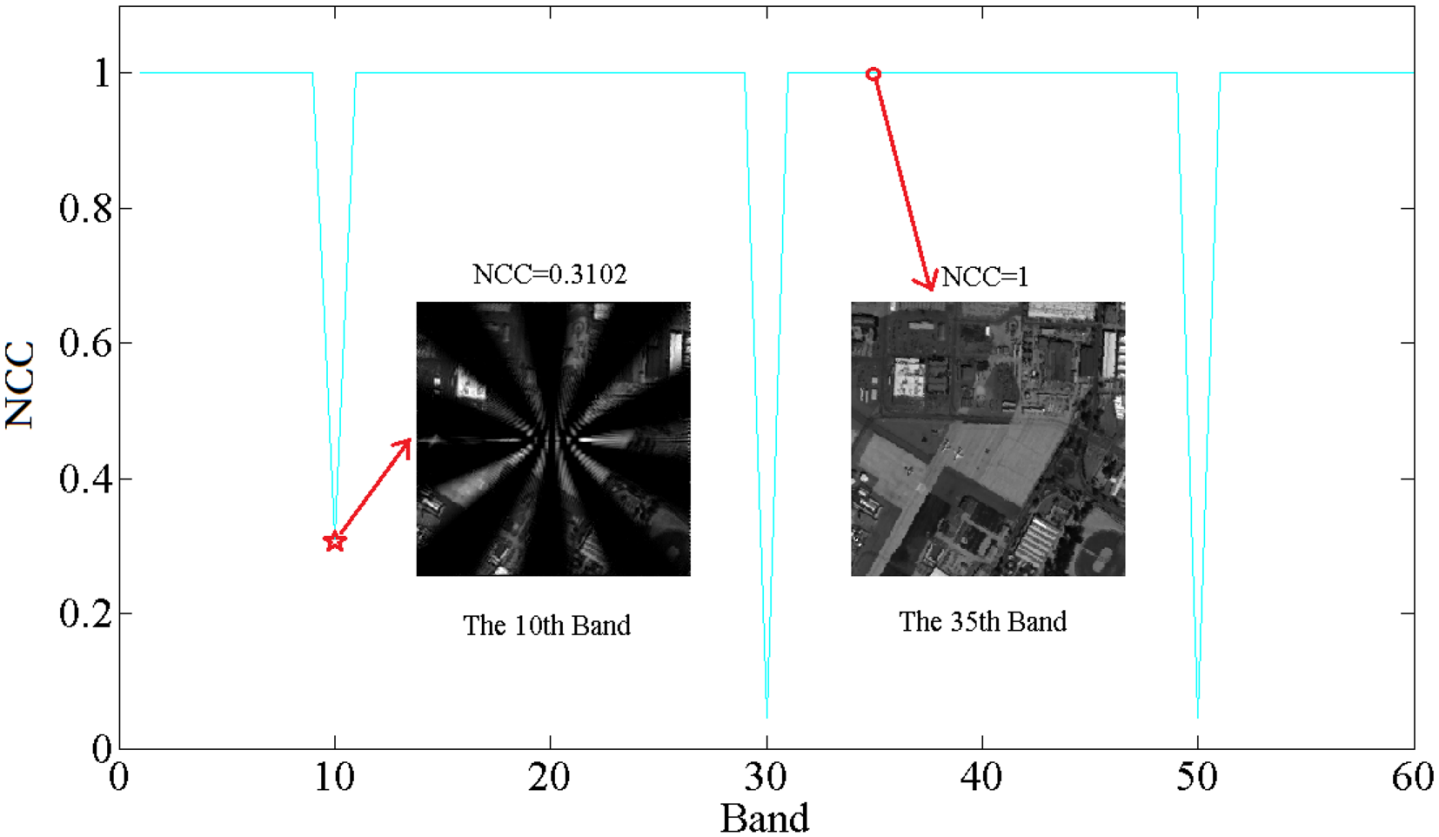
The NCC curve calculated by using the tampered random order $p$ in RSPF.

Another experiment is also considered to verify the security of the cryptosystem on protecting each band of the multispectral image. In this attack experiment, the random order $p$ controlling the number of singularities in RSPF is considered to be attacked. Since the order $p$ of each band in the multispectral is distributed by the chaotic sequence, the attack calculation is executed by using the tampered chaotic data. Suppose that the chaotic sequence is under protection except the 10th, 30th and 50th elements. The NCC curve between each band of the multispectral image and decrypted image is drawn and displayed in Fig. 9. It is easy to found that the NCC curve decreases dramatically for the tampered band image. The sharp curve demonstrates the validity of the extra key $p$. Some decryption results with tampered order $p$ are depicted in Fig. 9, in which the original information cannot be recognized. In calculation, the 10th, 30th and 50th elements (43.2745, 47.8555, 9.5471) are replaced by the values of the 20th, 40th and 60th band (33.6459, 20.0503, 4.1566). Consequently, the order $p$ of spiral phase controlled by the chaotic sequence can shelter the each band of the multispectral image well.

At the aspect of robustness analysis, some potential attack algorithms are employed to estimate the capability of the proposed cryptosystem on resisting the attack algorithms. During the processing and transmission, the encrypted data might be contaminated some unwanted noise. Therefore, the noise attack is executed first by using a noise model composed by Gaussian random noise with mean 0 and standard deviation 1. The mathematical definition of the noise model can be written as follows

12 $$ I^{\prime}(x{,}y) = I{(}x{,}y{\text{)[1 + k}} \cdot \sigma_{{0{,}1}} {(}x{,}y{)]} $$

where the function $I{(}x{,}y{)}$ and $I^{\prime}{(}x{,}y{)}$ denote the encrypted single band image before and after adding the Gaussian random noise, respectively. The coefficient b is the intensity factor for controlling the intensity of the noise. Besides, $\sigma_{{0{,}1}} {(}x{,}y{)}$ represents the random noise function with mean value 0 and standard deviation 1.

**Figure 10:**
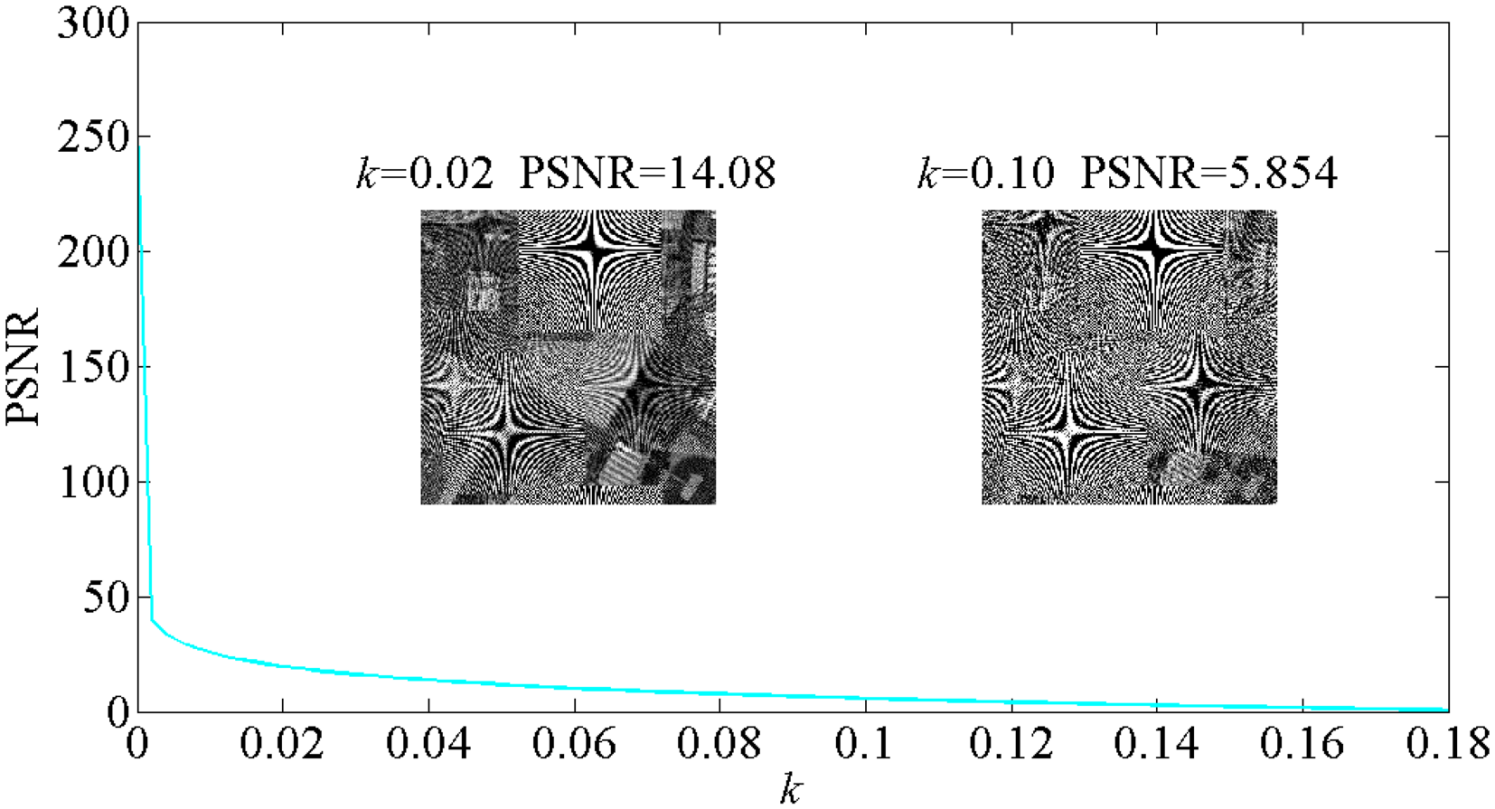
The experimental result of noise attack including the decrypted image achieved with k = 0.02 and k = 0.1.

Based on the noise model mentioned above, the noise attack experiment is implemented and the corresponding PSNR curve between the original image and attack results using variable noise intensity coefficient b is drawn and displayed in Fig. 10. In calculation, the attack calculation is performed for 91 times by using various noise intensity factor b from 0 to 0.18 with sampling step 0.002. As shown in Fig. 10, as the coefficient b increases, the PSNR curve keeps decreasing. Two decrypted image obtained by using k = 0.08 and k = 0.15 are shown in Fig. 10 and the outline information of the original image can be identified by human eyes. The results verify the proposed encryption system is robust against additive noise attack.

**Figure 11:**
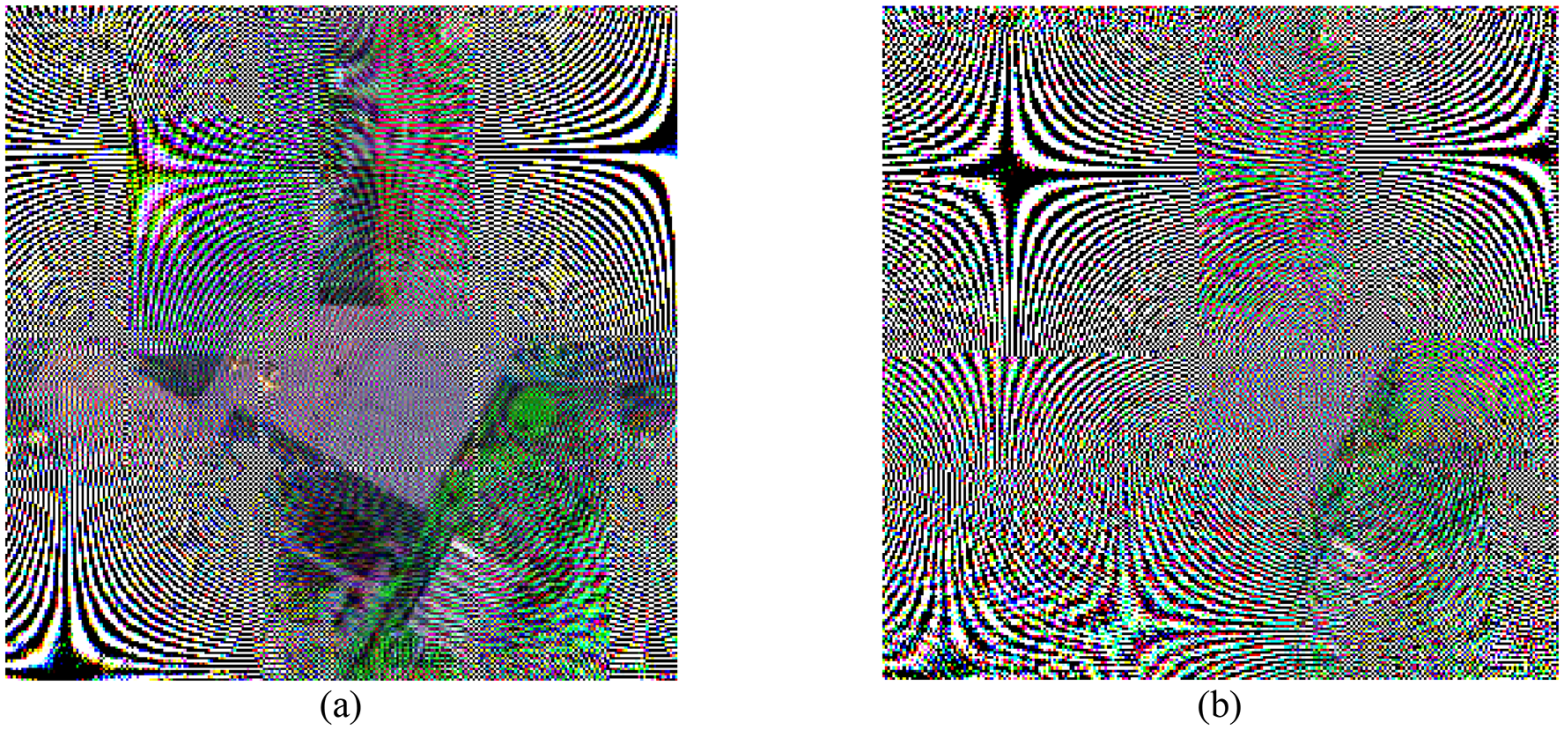
The test of occlusion attack: (**a**) the attack result of 1/16 occluded and (**b**) the attack result of 1/8 occluded.

The occlusion attack is also considered and executed to testify the robustness of the proposed encryption algorithm. In the occlusion attack experiment, the decryption process with all the correct keys is performed with encrypted data occluded partly. Here, both the 30th single band image and RGB color format are considered in occlusion attack and the corresponding occluded area are 1/8 and 1/16, respectively. Note that the occluded area of the encrypted data is filled by 0 in calculation and the corresponding single colorful occluded ciphertext and decrypted image are illustrated in Fig. 11a, b, respectively. As shown in Fig. 11, with the increasing of the occluded area, the quality of the recovered image decrease gradually. The outline information of the retrieved image is clearly visible with noise. The experiment results demonstrate that the proposed encryption scheme for multispectral image is robust against occlusion attack.

For testing the capability of this proposed cryptosystem on resisting the known plaintext attack and chosen plaintext attack, the following encryption model is considered and utilized follows

13 $$ E(u,v) = \xi^{\alpha } \{ I(x,y)\exp {[}i \cdot \phi_{1} (x,y)]\} exp[i \cdot \phi_{2} (x^{\prime},y^{\prime}){]} $$

where the symbol ‘$\xi^{\alpha }$’ represent optical gyrator transform with rotation angle $\alpha$ as mentioned in "Optical asymmetric cryptosystem" section. Besides, the functions $\phi_{1} (x,y)$ and $\phi_{2} (x^{\prime},y^{\prime})$ are two random phase masks having the same size with the input image $I(x,y)$.

**Figure 12:**
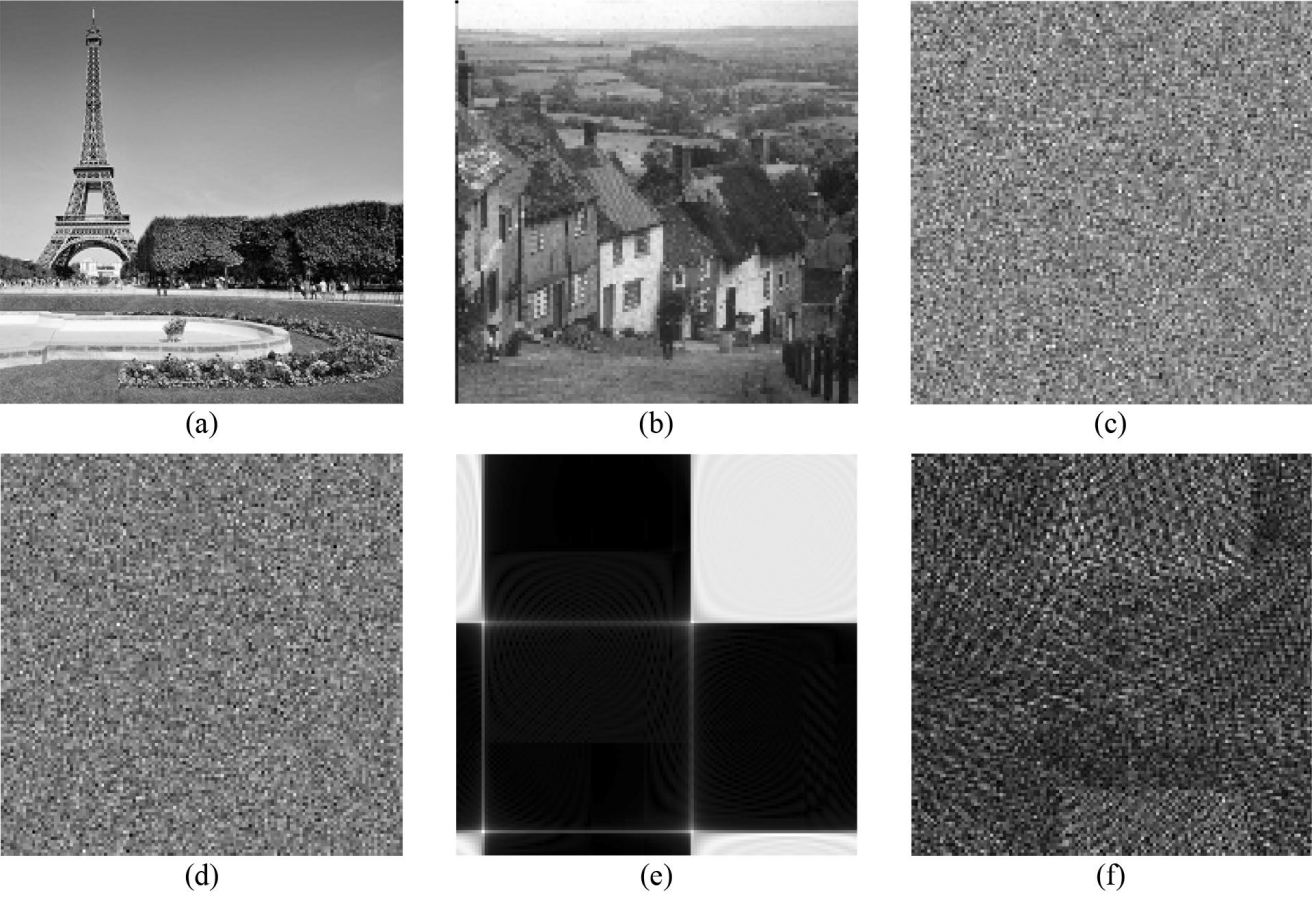
The known plaintext attack and chosen plaintext attack results: (**a**) the first test image, (**b**) the second test image, (**c**) the encrypted pattern of (**a**), (**d**) the encrypted pattern of (**b**), (**e**) the result of known plaintext attack and (**f**) the result of chosen plaintext attack.

In the attack experiment, the iterative phase retrieval algorithm and impulse function are executed to accomplish the known plaintext attack and chosen plaintext attack, respectively. For the sake of simplifying the calculation, two reduced test images ‘Cameraman’ and ‘Goldhill’ having $128 \times 128$ pixels are considered as two secret band images of the multispectral data to be attacked. Firstly, the test images are encrypted by using the proposed cryptosystem in this paper. The secret images and corresponding encrypted patterns are illustrated in Fig. 12a–d, respectively. In the attack calculation, we suppose that the original image ‘Cameraman’ shown in Fig. 12a and its encrypted data are obtained by the illegal attacker. Hence, the decrypted pattern depicted in Fig. 12d is attacked by implementing known plaintext attack and chosen plaintext attack, respectively. In calculation, the phase retrieval algorithm is performed with 2000 iterations in the known plaintext attack. Besides, the impulse function is executed for 16,384 times in chosen plaintext attack. Note that the encrypted data used in known plaintext attack and chosen plaintext attack is the ciphertext before the RMD. The known plaintext attack and chosen plaintext attack results are shown in Fig. 12e, f, respectively. Obviously, the decrypted images cannot be identified entirely and the secret information is well under protection. The noise-like pattern results indicate the high robustness of the proposed cryptosystem.

## Conclusion

We have presented an asymmetric optical image encryption algorithm for multispectral image based on random devil’ spiral Fresnel transform in optical gyrator transform domain. The proposed cryptosystem can be used to encrypted single band image, colorful image and multiple images according to different user. Firstly, the random devil’s spiral Fresnel lens phase is designed to encode the original image. Subsequently, the intermediate data are transformed by random devil’ spiral Fresnel transform in optical gyrator light filed and recorded by CCD. Then, the recorded image data divided into two masks by employing RMD in the asymmetric process. Finally, the final encrypted pattern can be achieved by scrambling with a random permutation matrix. For decryption, the ciphertext and private key are imported into the optical gyrator transform simultaneously. Some numerical experimental results have demonstrated the validity, security and robustness of the proposed encryption scheme.
